# Social Distancing and Social Biosensing: Intersubjectivity from Afar

**DOI:** 10.1007/s10606-022-09428-5

**Published:** 2022-07-26

**Authors:** Max T. Curran, John Chuang

**Affiliations:** School of Information, University of California Berkeley, Berkeley, CA USA

**Keywords:** Social biosensing, Computer-mediated communication, Intersubjectivity, Empathy

## Abstract

The shelter-in-place orders across the U.S. in response to the COVID-19 pandemic forced many relationships once sustained by in-person interaction into remote states through computer-mediated communication (CMC). Work, school, holidays, social engagements, and everyday conversations formerly experienced through rich and contextual in-person interactions instead have taken place on messaging, voice, and video chatting platforms that diminish or altogether lack many social cues and other qualities critical to social interaction. The difficulties feeling connected to one another observed during this period have stressed the need for novel forms of communication that enable deeper interactions. Social biosensing, the interpersonal sharing of physiological information, has shown promise facilitating social connection at a distance. In the present research we document the experiences of nine pairs of friends (N = 18) who navigated living through a shelter-in-place order, reporting on their experiences sharing their electrodermal activity (EDA) in response to short videos. Participants described the artificial and unnatural nature of communicating using typical forms of CMC and a range of interpretations of EDA as both emotional response and as representative of personal characteristics. We implemented a phased approach to study the temporal nature of forming an understanding of unfamiliar yet intimate data like EDA. Our results indicate typologies of meaning-making processes: “stablers”, “broadeners”, and “puzzlers”. We also interpreted our findings through the lens of intersubjectivity, analyzing how analogical apperception and dialogical interaction both play a role in participants’ meaning-making about their own and their partner’s biosensory information. We conclude with implications from this work pertinent to intersubjectivity theorists, social biosensing researchers, and CMC system designers and developers.

## Introduction

As social creatures, we strive to understand others and be understood ourselves. We relate to one another by communicating and learning about each others’ experiences and perspectives, shaped by personal contexts, histories, environments, and relationships. When describing an experience or feeling to a person in front of us we express a rich array of verbal and nonverbal cues, some consciously controlled and others unconsciously given off. Our word choices, facial expressions, physical gestures, vocal tone and volume, posture, and touch are all cues that make up our self-expression and, vice-versa, our ability to understand our interlocutor’s response and their own expressions. Coming to identify, understand, and even at times feel the inner states of others is a process of empathy.

The shelter-in-place orders across the U.S. and elsewhere in the world in response to the COVID-19 pandemic forced many relationships once sustained by in-person interaction into remote states through computer-mediated communication (CMC). Work, school, holidays, social engagements, and everyday conversations formerly experienced through rich and contextual in-person interactions instead have taken place on messaging, voice, and video chatting platforms that diminish or altogether lack many of the social cues and other qualities critical to social interaction. Digital communication, seemingly ever present and growing in prevalence and form, enables connection across space and time, freeing us of the necessity to be physically collocated with others, but costing us many of the qualities that contribute to a meaningful interactions. Concerningly however, empathy researcher Jamil Zaki describes technology as “our era’s biggest threat to empathy” in a recent book on the topic, further explicating that

“real-world conversation is rich and multifaceted; we catch a glint in a friend’s eye when he talks about his last date, a hesitation in his voice when he says work is great. We see excitement and hear doubt. Emotions are palpable and easy to share [...] online, social life is reduced to strings of texts and images.” (pp. 145–147) (Zaki, 2020)

Indeed, researchers tracking trait-level empathy have observed decreases over the last several decades, and at least correlational evidence of an association with technology use (Konrath et al., 2011; Konrath, 2013).

While social psychologists seek to understand the underlying effects of these active forms of online communication, many working in the fields of human-computer interaction (HCI) and CMC ideate, design, and assess innovative systems to augment or reconfigure our communications with one another even when we cannot be physically together as in this pandemic. In one vein of this work, biosensory information is collected from users’ bodies and behaviors and shared with others. These “social biosensing” systems are proposed for their potential to enhance communication via the inclusion of data about a person’s bodily responses corresponding to their felt internal experiences. Related work from social psychology and philosophy suggest many avenues to investigate the effects of sharing biosensory information. Previous research has indicated the difficult nature of grasping an exact meaning of biosensory information. Signals like EDA, heart rate, or EEG are, after all, imprecise indicators affected by a multitude of biological processes. Some of these processes are consciously felt and experienced like emotions, while others take place outside of consciousness such as the maintenance of bodily homeostasis for energy conservation and survival. This already high degree of inexactitude is further exacerbated by external factors of the recording device, environment, or behavioral artifacts like motion. On the other hand, the ambiguity of these signals can also serve as a boon. HCI and CMC literature has demonstrated that ambiguity can be advantageous for prompting pauses, reflection, and productive conversation about a phenomenon, which in turn can contribute more toward amicable mutual understanding than overinterpreted and absolute labels. Designing for questioning and contestability is particularly important for subjective experiences like emotions (Boehner et al., 2007). Yet, in order to make use of biosensory information there is still some meaning-making process taking place. It is crucial to have a preemptive understanding of this process in preparation for the move of social biosensing from research settings into broader use.

One particularly relevant conceptual framework to understand the meaning-making which takes place in social biosensing interactions is intersubjectivity, a philosophical, phenomenological approach to how two people come to agree on a concept through their individual perceptions and relations with one another. Ecological intersubjectivity focuses on the individuals in an interaction as separate entities who understand each other’s internal states by analogizing from their own previous experiences and perceptions. Radical intersubjectivity instead emphasizes the primacy and irreducibility of a social interaction between participants and propounds the significance of communication through language (Crossley, 1996). The philosophical perspective of intersubjectivity has been applied to CMC scenarios as well, lending support to the notion that complex conversational activity exists in spaces where social cues are reduced or reconfigured (Riva and Galimberti, 1998; Cui et al., 2013). Social biosensing has yet to be examined through this lens, which is the endeavor of this work. The sharing of EDA presents a particularly unique case for studying the intersubjective qualities of social biosensing because it is typically unfamiliar, in contrast to something with preexisting associations like heart rate, which allows for observation of people forming new meaning of an unfamiliar yet intimate bodily signal. This meaning may then be applied to understanding another’s EDA through ecological (analogical) or radical (dialogical) intersubjective means. The present research was designed to holistically examine how intersubjectivity plays out in social biosensing by implementing controlled exposure to one’s own EDA followed by one’s partner’s, as well as observing dialogue about their experiences with EDA between pairs of participants.

## Related Work

### Social Biosensing

Social biosensing extends individual biosensing via systems that employ physiological signatures of emotion-related bodily processes to facilitate interpersonal experiences involving the sharing and interpretation of sensor-driven social cues between people. Individual consumer grade biosensing, the detection, processing, and storage of physiological data about the body, is a growing market and, in turn, associated practice by users. The signals sensed by these devices can come in many forms including heart rate, accelerometry (motion), facial expression classification, electrodermal activity (EDA), and electroencephalography (EEG). Smart devices like phones, watches, eye wear, headphones, and hearing aids can embed many sensors within them that log sensed information for analysis by the wearer or use by a system to adapt the interface or functionality to match a user’s mental or physical state. Beyond individual tracking and sensing, and more germane to the present research, are efforts to understand the effects of sharing physiological data in interpersonal contexts from technological and psychological perspectives. Existing social biosensing work can be placed along a few different dimensions including: what type of biosensory information is used in the system (heart rate, EDA, EEG, etc.), how this signal is represented to participants (graphically, aurally, symbolically, etc.), and what communication medium it is employed in (e.g. augmenting in-person interactions or via CMC technologies).

Existing social biosensing research focused on heart rate suggests sharing of this signal can foster intimacy and prompt conversations. Heart rate has been the most common biosensory information examined in social contexts, represented in many different ways but often studied using qualitative methods aimed at understanding users’ first-hand experiences with a working prototype, either in-person or in CMC environments. While previously relegated to fields like affective computing in which physiological data is collected to be interpreted by algorithms designed to classify users’ emotional states (Picard, 2000), social biosensing research embeds these signals in person-person interactions. Studies of in-person systems like social virtual reality avatars with embedded heart beats (Janssen et al., 2010), movie watching with sound or visual cues of heart activity (Slovák et al., 2012), a park bench amplifying the heart beats of those sitting (Howell et al., 2019), or physical pulsing hearts held by interlocutors (Aslan et al., 2020), indicate the sharing of heart-based information can increase interpersonal closeness, intimacy, and connection. These findings seem to hold true in studies of sharing heart rate information via CMC systems as well. CMC systems that augment text-based messaging with heart rate have found associations of heart rate to emotional and psychological states like stress and assumptions that heart rate provided objective information about these states, making them more “real” (Liu et al., 2017b) and serving as prompts for asking how or what someone is doing (Hassib et al., 2017). One implementation of a smartwatch-based app incorporated heart rate into the more ambiguous and playful form of animated figures to be shared between participants found this form served as invitations to start conversations which involving collective meaning-making about their biosensory information (Liu et al., 2019b).

To explore social biosensing with a more ambiguous signal, the present research employs electrodermal activity (EDA), a measure of small changes in the moisture level of the skin with psychophysiological ties to emotional activation, rather than of heart rate. EDA is a particularly interesting signal for social biosensing because it lacks many of the priors associated with heart rate, and is more strongly associated with emotional activation (feeling relaxed vs. stimulated) which leaves room for interpretation of valence (negative vs. positive) (Boucsein, 2012; Micoulaud-Franchi et al., 2014). One of the earliest EDA sharing prototypes, the “Galvactivator”, was a glove that both measured EDA and adjusted the brightness of an embedded LED light based on the EDA levels. Over 1,000 of these devices were built and given to audience members in a large lecture hall, and by monitoring the cumulative brightness in the room researchers were able to map high and low arousal and engagement periods like brightening when new speakers coming on stage, or dimming when a speaker had been talking for a long time (Picard and Scheirer, 2001). Another light-based in-person EDA sharing prototype study was “Moodlight”, a color-changing lamp emitting cooler colors for lower levels of arousal via EDA and warmer ones for higher levels. The system interestingly served as both encouragement of self-disclosure and a reflection of its calming effects, though the authors point out the discomfort some participants felt about externalizing an uncontrollable display of their inner states and a lack of questioning regarding its accuracy representing their emotions (Snyder et al., 2015). In their work with EDA-based color-changing fabrics woven into shirts, Howell and colleagues sought to embrace the ambiguity of EDA and the slow and subtle color shifts of the heat-activated textiles used in their designs. The authors intended to prompt critical reflection of capturing emotions in an EDA sensor, designing explicitly for ambiguity and contestability, however, as was the case with Moodlight, participants sought validation by way of the color displays and were faced with insecurities when it did not match what they subjectively were feeling, interestingly trusting the display of the data over their own self-knowledge (Howell et al., 2016, 2018). Few studies have looked at EDA sharing in CMC scenarios. Smaller user studies of prototype systems like modifying the font size in a messaging application to affectively augment the communicator’s written text (DiMicco et al., 2002), or modulating blur effects or haptic vibration alongside recorded videos (Shirokura et al., 2013), find that representations of EDA can convey a sense of excitement. Indeed, a review including many of these examples of social biosensing notes the potential of displaying normally hidden cues like physiology to improve social interaction and connection (Chanel and Mühl, 2015).

Unlike other signals, the relative obscurity of EDA fosters a sense of ambiguity which is purportedly a key quality in maintaining user agency in the design of social biosensing systems. Heart rate in particular as a social signal tends to have pre-existing associations with heart-based signals like intimacy and anxiety. In an experimental investigation of implicit associations with heart rate information, Merrill and Cheshire found that when the heart rate of participants’ study partners were displayed as elevated they rated their partner higher in anxiety and cooperated less often. Interestingly, in another condition using a fictional elevated signal there was no change in cooperation. The researchers make the case that for unfamiliar signals (or even fictional ones) system designers hold a lot of power enabling them to “teach” users associations with these signals, whether for better or for worse (Merrill and Cheshire, 2017). It may be that the uncontrollable nature and scientific or medical status of biosensory information engenders it with a sense of objectivity and truth. A relevant study lending evidence to this point documented the effects of what the authors dubbed “neuroenchantment” in which most participants, even those who had studied neuroscience and psychology, were convinced that a mock brain scanner could read their complex inner thoughts (Ali et al., 2014). This afforded authority can be dangerous, especially considering the relative lack of convergence in psychophysiological research regarding signatures of occurrence of particular emotions. In contrast, some HCI researchers propose strategies like embracing ambiguity to foster discussion, interrogation, and interaction with information conveyed by a system (Gaver et al., 2003) as well as allowing for and encouraging questioning and contestability (Hirsch et al., 2017). Specifically regarding emotional data and in line with earlier discussed philosophical perspectives like intersubjectivity, Boehner et al. makes a case for modeling affect as interactionally constructed rather than as codified information to be extracted, and for the recognition and incorporation of the limits of both objective and subjective conceptions of emotions (Boehner et al., 2007). In terms of how to represent these signals, one investigation comparing different visual representations of biosensory information found that participants preferred more raw forms of data visualizations like graphs over interpreted representations like emojis because they conveyed richer information allowing them to draw their own conclusions (Liu et al., 2017a).

### Empathy and Intersubjectivity

Empathy is the prime social process taking place in social biosensing scenarios, thus application of sociopsychological empathy research can illuminate much about the underlying mechanisms at play. Social biosensing posits the conveyance of some quality of one’s internal experience to another by revealing bodily information, and the interpretation of another’s inner states in this way engages a process of empathy. Empathy was initially conceptualized simply as a trait that varied among individuals, wherein some people are just naturally more empathetic than others. While there may be dispositional and automatic *components* of empathy (Davis, 1983; 2006), recently a strong case has been made for the situational and even consciously regulated nature of empathy. Zaki’s theory of motivated empathy puts forward the idea that empathy is in fact mutable. The theory describes three opportunities for motives to influence the process of empathy and strategies that may be employed at each: approaching or avoiding a potentially empathy-eliciting situation, increasing or decreasing attention to emotional cues, and deciding how to appraise these cues (Zaki, 2014). Cognitive empathy in particular involves an observers’ awareness and identification of target person’s internal states without the observer necessarily feeling those states. The core elements at play in an empathic interaction are emotions which involve subjective, behavioral, and physiological factors. EDA for example is one physiological component tied to the arousal or activation dimension of emotion, and thus may serve as a useful tool that can be technologically captured and wielded to encourage cognitive empathy.

A philosophical substrate of empathy is intersubjectivity, a phenomenological approach to how two people come to agree on a concept through their individual perceptions and relations with one another, and by engaging with intersubjectivity and its phenomenological roots the present work aims to interrogate social biosensing more deeply. With its focus on perception (of the world, including other people) via the body, the philosophy of phenomenology attributed primarily to Edmund Husserl and expanded upon by others like Martin Heidegger and Maurice Merleau-Ponty is highly pertinent to social biosensing. Research employing phenomenological methods asks questions like “What is it like to experience a particular situation?” through qualitative methods such as in-depth interviews (Bloor and Wood, 2006). Phenomenology describes simulation of direct experiences as the way we learn about and are able to act in the world around us (Merleau-Ponty et al., 1964). A key component of phenomenology is its positioning of perception as *intentional*; rather than passively receiving information about the world, phenomenologists argue that our minds “reach out towards” other entities resulting in perception. Intersubjectivity applies this philosophy to the social realm. In his book *Intersubjectivity: The Fabric of Social Becoming*, Crossley describes intersubjectivity as the notion that our experiences are neither wholly internal and constructed nor does the world exist in some pure stable state irrespective of our interactions with it (Crossley, 1996). Consistent with phenomenological views of the irreducible nature of our interactions with the world, intersubjectivity carries this status to interactions with one another. From an intersubjective perspective, meaning in social contexts is a shared understanding created from one’s reference point and is socially mediated throughout an interaction (Given, 2008). Work engaging with intersubjectivity in CMC scenarios has employed the concept to re-imagine CMC systems as rife with complex coordinated activities enabling the activation of a social relationship beyond simply transferring information (Riva and Galimberti, 1998). In later discussions of social presence in CMC, intersubjectivity constitutes the highest level of presence representing a move beyond awareness of others’ internal states into the sensation of a mutually constructed social space (Cui et al., 2013).

Intersubjectivity is particularly germane for understanding empathy and social biosensing, as these technologically sensed signals are perceived in one form or another by the owner of the body as well as another person. Crossley interprets and synthesizes several philosophers’ takes on intersubjectivity (Crossley, 1996). From Husserl, considered by many to be the founder of phenomenology, Crossley quotes:

“Empathy is an imaginative process [...] the other has a body which is identical to mine and they move as I do, my body and movements embody conscious life and experience, therefore the other’s probably does too. [...] One will continue to believe that the other is a conscious subject and to imaginatively transfer experiences onto them for as long as they continue to behave in a way which is understandable from the point of view of a conscious subject.” (p. 6) (Crossley, 1996)

While social biosensing was not in the purview of either Merleau-Ponty, Husserl, Crossley, the predication of empathy on evidence of another’s body clearly resonates with the position of social biosensing research. As seen in related work, the bodily sourced signals in social biosensing interactions can aid in social presence (Slovák et al., 2012), intimacy (Liu et al., 2017b), emotional perspective-taking and interpersonal closeness (Liu et al., 2019a), and cognitive empathy (Curran et al., 2019). Furthermore, self-projection from one’s own biosensory activity (or conjectures regarding what it would be) has been observed in anonymous contexts as well as the present study with friends. A Husserlian perspective on intersubjectivity and empathy includes a process dubbed “analogical apperception”. Apperception itself is what allows us to perceive for example an entire building’s presence from only its facade, perceiving more than we actually see based on prior experience with buildings. With other people however, this apperception can only be analogical as we do not have direct access to the experiences of others as we do our own experiences of a house. Thus, the Husserlian logic of empathy is that if I observe another with a body like my own, and that my body and movements are indicative of conscious life and experience, then the other’s body must be as well (Ricoeur, 1967).

What has been described so far is what Crossley dubs “ecological” or *analogical* intersubjectivity, and is a process primarily occurring and observed within an individual or the projection of an individual’s experiences into another. Drawing on the works of Maurice Merleau-Ponty, “radical” or *dialogical* intersubjectivity is distinct from the ecological model. Radical intersubjectivity shifts the focus from the individual to the dialogical situation which unfolds among people in an interaction, treating this as the basic unit of analysis. This perspective emphasizes that “perception is always a view *from* somewhere, a view from “nowhere” is incoherent”, indicating the impossibility of separating meaning from the full context in which it is generated. Unlike the ecological model, radical intersubjectivity accounts for language and communication between individuals in a social interaction and embraces these tools as integral to the process of empathy. Both ecological and radical intersubjectivity approaches are relevant to social biosensing scenarios in which signals are exchanged which each person may produce individually and have previous experience with from which to project, but are also subject to conversation filling in the gaps characteristic of any biosensory information.

## Research Methods

This research was aimed at gaining a more qualitative understanding of the sharing of EDA data between pairs of friends. A fundamental component of this study stemmed from the shelter-in-place conditions resulting from the COVID-19 pandemic under which data collection was carried out. This created an unfortunate but natural opportunity to study communication between close pairs who could suddenly only interact at a distance. In order to better understand this new context and its powerful effects on maintaining social connection, and whether social biosensing could supplement this communication to feel closer to in-person interactions, the first research question this study seeks to address is:

**RQ1:** (i) What are the experiences of newly physically distant friends with computer-mediated communication, and (ii) in this context, what are their perceptions and responses regarding sharing EDA information?

Within this established context, this study also sought to understand the dynamic and temporal role social biosensing may take in this new reality. One form of digital interaction that rose following SIP orders was sharing video content on social media, messaging, and video calling platforms. This interaction in which interlocutors bond over their own and their friend’s emotional responses to content of interest can occur asynchronously by sharing the content in a message thread for a friend to react to, or synchronously by watching the content together on a call. These kinds of synchronous interactions at a distance can be helpful to stave off loneliness and feel connected in a time of forced physical separation (Nault et al., 2020). This study models and builds upon this scenario by using video content to elicit EDA responses in participants to interpret and share. Additionally, in previous social biosensing work, people have tended to view others’ EDA responses in comparison to their own, whether or not they had ever observed their own EDA. Therefore the goal of this study was to understand the meaning-making process between close pairs around unfamiliar biosensory information, and to examine the separate stages of seeing one’s own EDA response and then subsequently another person’s to the same stimulus. Thus, the second research question of was:

**RQ2:** How do people form understandings of unfamiliar biosensory information (EDA) through observation of their own EDA, a known other’s EDA, and through conversation?

### Study Procedures

**Fig. 1:**
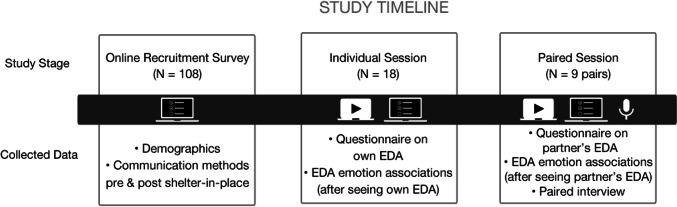
Study procedures timeline.

Figure 1 summarizes the study procedures. Participants first completed a set of online questionnaires primarily intended to recruit for the later stages of the study. The questionnaires asked demographics questions as well as questions about the communication methods and behaviors between the participant and a close other (e.g. a friend, romantic partner, family member, etc.) before and after the area’s shelter-in-place order.

From the online questionnaires, participants who indicated interest in continuing in the study and who listed a partner who did not live with them and who also completed the online questionnaires were contacted with an invitation to participate in the individual session. In the individual session each participant’s EDA was recorded using an Empatica E4 while they watched a set of online videos of their choice via YouTube. While instructing each participant in putting the EDA sensor wristband on, the researcher gave a short description of EDA to the participant which read: “EDA stands for electrodermal activity which measures the moisture levels of your skin. EDA can be related to your emotional responses.” This definition was used for accuracy while being intentionally vague and not overly authoritative in order to allow for participants to assign their own meanings to the signal. This also ensured a shared baseline understanding of EDA for all participants, none of whom reported previous experience or knowledge of EDA. As described further in the findings below, some participants referred back to this definition while others did not. The EDA information from the E4 was displayed as a live, full screen, real-time graph on a tablet placed next to the screen playing videos.

After the wristband was secured, a 2-minute seated rest period began. This period allowed for the EDA signal to stabilize and participants to see their EDA with no video stimuli. For the first video they watched, participants selected from a predetermined list of short 5-minute videos which was curated by the researchers to be of interest to participants and elicit a range of emotions while adhering to the bounds of ethical research by avoiding disturbing or overly extreme stimuli. This list of videos included: a small dog being groomed, a comedian’s description of mental health care, a pop music video, an impassioned speech about climate change, a clip from a documentary about people with strange addictions, a commencement address by a known figure, a trailer for a documentary about record breaking rock climbing, among others. If a participant’s partner had already completed their individual session, the first video they watched was selected for them to be the same as the one chosen by their partner. This ensured that participants could later compare their subjective experiences and EDA activity with their partner’s response to the same content. After watching this first video, all participants watched a second video of their choice on YouTube while observing their EDA. Some participants chose another video from the existing list, while others found other videos to watch. This was done to allow participants a chance to more freely explore and form meaning around their EDA response. Following watching both videos, participants answered a series of questions, both open response and multiple choice, about their impressions of seeing the EDA data and how it related to their experience watching the videos, why they selected their videos. Participants also rated the degree to which they felt each of 27 emotions (Cowen and Keltner, 2017) was related to changes in the EDA information on 7-point likert-type scales from “Not at All” to “Extremely”: admiration, adoration, aesthetic appreciation, amusement, anger, anxiety, awe, awkwardness, boredom, calmness, confusion, craving, disgust, empathic pain, entrancement, excitement, fear, horror, interest, joy, nostalgia, relief, romance, sadness, satisfaction, sexual desire, and surprise.

**Fig. 2:**
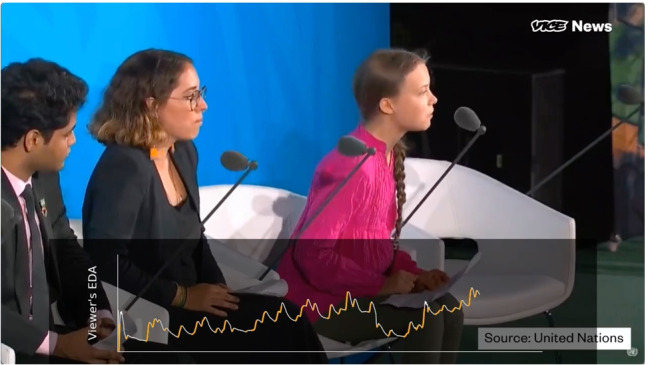
Sample screenshot of what a participant would see of their study partner’s EDA. The time series graph along the bottom filled in as the video played and sustained positive slopes in the EDA data are highlighted in orange.

Once both participants in a given pair completed the individual session they were invited to participate in a paired session with their partner conducted over the Zoom video calling platform. In this session participants first watched a composite video of their partner’s recorded EDA activity played as a moving graph alongside the first video which they had both watched. Figure 2 shows an example screenshot of a what a sample participant saw of their study partner’s EDA.

After seeing their partner’s EDA response embedded in the video, participants responded to the same questions they answered about their own EDA information, but this time about their partner’s EDA information and their partner’s experience. This included a second round of rating their perceived associations of changes in EDA with each of the 27 emotions using the same scales as previously. This paired session concluded with a group interview covering pairs’ communication behaviors while separated and their experiences and perspectives on interpreting EDA information about their study partner. This was conducted in pairs in order to focus on the relationship between each of the two friends, allowing for both participants to answer interview questions, talk between themselves to confirm or build on responses, and trigger elaboration and inspiration by hearing each other’s responses. As these participants were purposefully recruited as pairs of friends, it was not a concern that responses would be unduly swayed by the presence of their partner.

An effort of this particular study’s design was to address how people’s understanding or assigned meaning to EDA changes over time. Specifically, the phases of this understanding examined in this study were: 1) each participant seeing their own “live” EDA information via a moving graph while watching short videos they had selected, 2) seeing a friend’s recorded EDA information as an embedded graph in a video they watched (chosen such that it was a video the participant watched), and finally 3) a group interview with their friend, some of whom saw an additional video with both their own and their friend’s graph embedded in the content for comparison. Participant answers to open response questions were collected after parts 1 and 2, whereas part 3 insights were drawn from the interview transcripts. These phases were designed to serve as a proxy for real-world scenario where social biosensing becomes proliferated into wider use in which a user would record their own data, see the data of close others, and talk about the data and practice amongst their friends.

The bulk of the analyzed data in this study were qualitative, consisting of the paired interviews and individual open responses to questions administered after participants had seen their own EDA information and their study partner’s. These data were inductively coded by two researchers in two rounds of coding. Researchers specifically applied descriptive, process, and in vivo coding methods to the data (Saldaña, 2015). These codes coalesced into themes which became the set of findings presented in the results, including illustrative quotations from interviewees. In addition to the open responses questions, the EDA emotion association questions were used to look at individual profiles of how participants’ internal models of EDA information changed between seeing their own and then seeing their partner’s EDA information. Some descriptive analyses were also conducted on the online questionnaires to assess changes in communication behavior before and after the March 2020 shelter-in-place order.

## Results

### Socially-Distant Socialization and Perceptions of EDA

This study took place during the unique circumstances of the COVID-19 global pandemic and resultant shelter-in-place orders in California. Results related to RQ1 (i) allow for a better understanding of the communication ecosystem study participants were experiencing at the time of data collection and their reactions to sharing and receiving EDA information.

108 responses to the online recruitment survey were received and 88 of these respondents reported not living with the close other they answered questions regarding. The mean age of these 88 respondents was 22.259 (standard deviation 3.110), 69 self-identified as female with the remaining 19 identifying as male. When answering questions about a close other person, 67 respondents described the person as a friend, 13 as a romantic partner (partner, boyfriend, girlfriend, spouse), 5 as a family member (family, parent, sister), and 2 as other or no response. Respondents were asked to rank their frequency of using six different methods of communication: face-to-face, video call, text message or messaging app, over the phone, by mail, and other from most to least frequently used, both before and after the SIP order. Looking at those 88 who did not report living with their close other, “face-to-face” frequency decreased the most after the SIP order compared with before. Video calling and text messaging had the greatest increase in frequency, followed by phone calls, mail, and other.

From these 88 respondents, 9 pairs (N = 18 total) of friends who did not live together accepted an invitation to participate in the follow-up sessions which constituted the main portion of the study. These sessions included a group interview with questions about how their communication with one another had been affected by the SIP order, and what their experiences communicating with each other had been during the pandemic. All participants were located in the US, living at least part of the year near a large west coast public university, and spoke fluent English. Other characteristics of these 9 pairs are shown in Table 1.

**Table 1:** Self-reported characteristics of the 9 pairs of friends who participated in all phases of the study.

Pair	Genders	Ages	Length of friendship	Primary communication in SIP
A1/A2	F/F	26/25	1–5 years	Messaging, video calls
B1/B2	F/F	22/22	1–5 years	Messaging, some face-to-face at work
C1/C2	F/F	21/21	1–5 years	Messaging, phone calls
D1/D2	M/F	20/20	6 months–1 year	Messaging, phone calls
E1/E2	F/F	21/22	1–5 years	Video calls, texting
F1/F2	F/M	26/24	5–10 years	Phone calls
G1/G2	F/M	20/19	6 months–1 year	Messaging, phone calls, video calls
H1/H2	F/F	23/23	1–5 years	Video calls, messaging
I1/I2	M/F	27/27	1–5 years	Phone calls, messaging

When asked about their communication behaviors with one another during SIP, participants voiced many of their frustrations with CMC technologies. Although some common likes or advantages came up including acknowledging the convenience, appreciating the flexibility afforded by asynchronicity, and it’s higher degree of control with respect to self-disclosure. Comparatively many more words and time were spent on participants’ disappointment regarding CMC. These responses largely confirm long-standing knowledge of CMC technologies studies demonstrating their status as insufficient and unfulfilling substitutes for in-person interaction. Though CMC serves as an important supplement to these relationships for its advantages like asynchronicity, convenience, and control of self-presentation, it has yet to meet the standard for replacing face-to-face meetings. Latency and interruptions from device malfunction, a lack of important interactional qualities like real-time responses and awareness of others’ emotional cues and behaviors, and feeling locked-in by the limited capacity of CMC systems are significant hurdles for CMC researchers and developers to overcome. Interestingly, when asked how participants understood their friend’s reaction to something shared asynchronously such as a message or post on platforms like Facebook or TikTok, answers like “I can picture it”, “I can hear their voice”, or “I can imagine them snickering at their phone” were common among all interviewed pairs.

#### Initial Impressions of EDA

What role might EDA play in the context of these problems posed by CMC that complicate feeling connected when physically apart? RQ1 (ii) sought to address this by investigating the interpersonal processes of social biosensing in distanced communication between friends. In previous research, social biosensing has been posited as a novel social cue, affording qualities like intimacy and cognitive empathy which can be difficult to convey over CMC. Conveniently, biosensory data can be transmitted and displayed over distance, but how people would react to this unfamiliar cue is unclear. We can begin by examining how participants responded to seeing their own biosensory data and their study partner’s across the different stages of the study.

Results indicated that participants were roughly split on whether their EDA information behaved how they expected it to, but nevertheless participants reported high interest in observing both their own and their study partner’s EDA information, with slightly more interest about their partner’s over their own. Although this study did not include enough participants to make quantitative conclusions, there was a notable trend for participants to associate EDA more with arousal-related emotions like interest, entrancement, amusement, and boredom upon observing their partner’s EDA information compared to when they had seen their own.

**Fig. 3:**
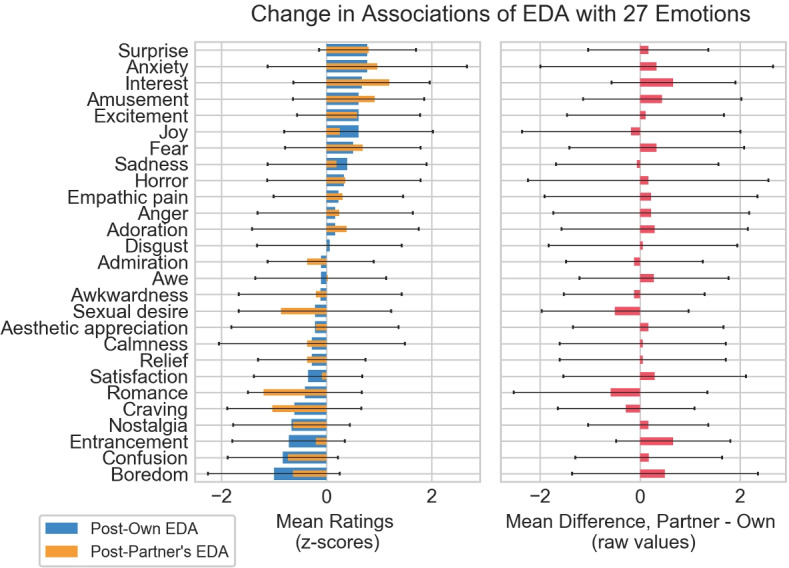
Participant’s (N = 18) rated impressions of EDA’s association with 27 different emotions at two time points: 1) after seeing their own EDA and 2) after seeing their partner’s EDA response, plotted as intra-individual z-scores (left). Changes in raw association ratings between these two time points are also shown as a difference score (right). Error bars represent one standard deviation.

As part of the questionnaires administered both after participants observed their own EDA and after they saw their study partner’s EDA, participants responded to several multiple choice rating scale questions. 11 out of 18 participants reported their own EDA information changed as they expected it to, and 13 out of 18 participants said the same about their study partner’s EDA data. Participants also reported a slightly higher degree of interest in their study partner’s EDA information than their own. In addition, participants were asked to rate how much they associated changes in EDA with each of 27 different emotions sourced from Cowen and Keltner (2017). Responses to these scales were collected once after participant’s saw their own EDA information, and then a second time after viewing their partner’s. Figure 3 depicts responses to these emotion ratings at these two time points.

An important note here is that the sample size is low to draw conclusions from this kind of quantitative analysis, evidenced by the high standard deviations. Additionally, although both participants in a given pair watched the same video, which video this was varied between pairs and as described later in these results participants reported that the content of the video limited what they could interpret from the EDA information. Despite these caveats, this visualization is an illustrative representation of how participants’ meanings of EDA changed between the two time points. Emotions like entrancement, interest, amusement, and boredom appear to have been attributed more to EDA after seeing a friend’s EDA information which are emotional states associated with high or low arousal.

In summary, participants reported significant problems and drawbacks using CMC to maintain social connection at a distance, some of which social biosensing may be poised to ameliorate such as conveying emotional cues which participants currently report imagining in lieu of seeing or hearing directly. With regards to EDA in particular, participants were highly interested in observing their own and partner’s EDA responses, and their associations of EDA to different emotional states seemed to shift after seeing their partner’s EDA data. Critical to understanding the outcomes of this kind of system in the wild is the question of how people create and update a meaning for previously unfamiliar biosensory information when observing their own and someone else’s. The next section utilizes the qualitative data collected in this study to delve into this question.

### Meaning-Making with EDA

The remainder of the results of this study seek to address RQ2 by unpacking precisely how participants came to assign a meaning to EDA information over the course of the study, what role EDA played in interpreting their partner’s experience, and what kinds of use cases socially sharing EDA could fill. The occurrence of analogical as well as dialogical intersubjectivity can also been seen in these results by

#### Temporal Meaning-Making Typologies

In addition to the limited quantitative analyses above, open responses and interview data further illuminated how participants’ conceptions of EDA information changed over time with additional exposures. The questionnaires administered after exposure to their own as well as their partner’s EDA information included open response questions asking participants to describe how the EDA information related to [their/their partner’s] experience watching the video, whether there were times when [their/their partner’s] EDA information changed in an unexpected way, and whether they learned anything about [their/their partner’s] experience from seeing the EDA information. From participant individual responses to these questions, as well as analysis of the group interviews, a typology of temporal changes in understanding of EDA emerged. Using a lens of intersubjectivity, these typologies indicate patterns in meaning-making about EDA in a social context via observation of another’s EDA and through conversation about EDA with their partner in the paired interview. An additional noteworthy point is that it was not necessarily the case that both members of a given pair fell into the same typology, in fact this was only the case for 4 of the 9 pairs.

*Broadeners* was the first of these typologies, which were those who broadened their definition of EDA over the course of the study. The 6 of the 18 participants who fell into this category began with one or more inner states such as “stressful emotions” or “enjoyment” they felt corresponded with changes in their own EDA as they had watched the videos. After seeing their partner’s EDA, participants of this typology expanded the set of triggers they believed EDA to take, either by adding similar or related states e.g. “calm” as the opposite effect of “stress”, or reducing the specificity of the states like moving from “negative emotions” to “strong emotions”. In the final stage of the group interview, these participants tended to agree with their partner’s assessment of the correlates of EDA and incorporating these to their own model of the signal. A1 was a good example of a broadener, beginning with a concise association of EDA with stress after seeing their own EDA information and then expanding to association with “the emotional parts” of the video:

“when I watched mine I thought it was more like stress but then after doing hers I’m more like eh well that wasn’t really stressful, but like it just went down [...] so I guess that’s why I changed it to more emotional rather than just stress” - A1

Participants in this group highlighted the role of dialogical intersubjectivity in which meaning was created within the interaction between the participant and their partner, not solely from their own individualized interpretations.

*Stablers* was next most common typology, comprised of those who were fairly stable in their conceptions of EDA throughout the phases of the study. The 5 participants in this typology typically started with an understanding of EDA that wasn’t tied to particular emotions but rather general patterns of inner states such as EDA as indicate of “emotional ups and downs”, “following the intensity”, or simply a “response” to the content. With exposure to their partner’s EDA as well as the conversation with their partner in the group interview, these stablers interpreted new changes through their existing definition, seeing specific emotions as instances of their more abstract existing conceptions of EDA’s meaning. E1’s descriptions of their understanding of EDA over the course of the study nicely illustrates this typology. After their first session seeing their own EDA E2 wrote:

“It seems to **reflect what parts of the video are most emotional to me**, since the EDA noticeably changed when I switched to a more serious video” - E2

After seeing their partner’s EDA response to the same video they watched, E2 noted particular emotions like shocking and exciting, but maintained a more general underlying understanding, stating:

“The EDA definitely peaked when there was a shocking or exciting piece of the video. It generally had a downward trend as the video went on but **peaked during certain points**.” - E2

And finally in the group interview, E2 maintained the inclusion of shock or interest, but functionally kept with her original more abstract understanding akin to “significance”:

“I did see [my EDA] kind of waver up and down and then it would kind of peak any time something interesting came up or shocking and the same when I watched Veronica’s video, I think the EDA changed color at one point whenever there was a peak maybe to **denote that it was significant**.” - E2

This group of participants were most representative of analogical intersubjectivity, wherein participants created a meaning from observing their own EDA information and analogized their partner’s experience using this maintained definition.

*Puzzlers* was the final typology that emerged in temporal qualitative analyses. This set of 4 participants experienced growing uncertainty and skepticism with further exposure to and discussion of EDA. Participants of this type either began and kept with the notion that EDA is “random” has “no correlation” with emotion, or at times held associations with particular emotional states but were contradicted by later events. In either case, by the end of the study these participants maintained a sense of confusion or puzzlement. Notably, participants in this category tended to be those who at some point in the study noticed or suggested EDA could be affected by non-emotional, external factors like temperature or the tightness of the wristband. One participant who fit this model well was E1 who started out with a sense of skepticism that only solidified as the study progressed. In the questionnaires following the first session with her own EDA displayed, E1 wrote:

“**I can’t say I have enough evidence to make a conclusive statement**. I guess it felt like my EDA information could fluctuate quite rapidly. [...] I think when I felt cold, my EDA levels increased” - E1

After viewing their partner’s EDA information, E1 had a slight notion of a stable meaning but maintained a degree of doubt regarding the connection between watching a video and EDA activity. In the group interview, E1 summarized her view that she felt EDA was inconsistent and affected by too many factors to make a strong statement:

“if you’re asking me what factors affect it, what increases or decreases it, **I think there’s just a lot of variables at play** and especially like seeing [E2] talk about my reading versus what I saw in her reading like **there’s not really any consistency** per se, so yeah.” - E1

As these participants did not assign a particular meaning to the EDA information, intersubjectivity played less of a role without the need to engage with a particular meaning. A few described further confusion when hearing their study partner’s interpretations of EDA which could be an example of dialogical intersubjectivity, but whereas “broadeners” used their partner’s perspective to modify their own, for these “puzzlers” it fueled their continued skepticism.

The remaining 3 participants did not fall neatly into any of these typologies and, importantly, are indicative of the inexhaustive quality of these findings. Two of these participants seemed to change their idea of EDA significantly at each time point of the study, and the other neither articulated an idea of EDA’s meaning nor stated uncertainty in the interview. These and what are sure to be others are avenues to further understand in subsequent research.

These patterns in participant responses suggest an intersubjective account of EDA in social contexts. EDA took on new meanings from participants’ experience with it expanded from observations of their own EDA responses, to exposure to those of a close friend, and finally in comparison and active conversation with their friend. Furthermore, the greatest magnitude and clarity of change seemed to occur when participants were in discussion with their study partner in the group interview, suggestive of the power of communication and exchange with others with regards to meaning-making of an unfamiliar signal. Intersubjectivity and its significance for understanding social meaning-making of EDA is delved into further in the Section 5.

#### Roles of EDA

Outside of the temporal framework, further analyses of the in-depth group interviews using emotional, values, and in vivo coding techniques revealed two distinct roles or functions EDA played in the process of interpreting their study partner’s internal states: EDA as *emotion* and EDA as *persona*.

##### EDA as Emotion

By utilizing emotional coding one notable outcome was the wide range of emotional words or other references to inner states participants used when describing EDA. From interview transcript analysis, approximately a third of the instances where participants referred to an emotion word when describing EDA fell into a a set of specific, valenced emotional states e.g. “funny”, “fear”, “excitement”, and “disgust” and the other two thirds as a set of broader terms relating to more general arousal: “interest”, “investment”, “attention”, or “strong emotions”. This breakdown is in line with the common typology of “broadeners” described in the previous section, where many participants ended up with a more encompassing understanding or personal definition of EDA that more closely aligned with general arousal than specific emotions. Although participants viewed a list of emotions when responding to the associations questions, this list spanned a very wide spectrum of emotion words and thus was not a likely source of priming of one type of emotion over another.

A notable proportion of pairs, 4 of 9, had some conversation about how their ability to assign meaning to the EDA was limited by the emotional range of the video content they watched. B1 described this nicely when reflecting on the dog groom video she and her partner watched and saw their EDA responses to:

“I feel like it depends on the video. If it is something that’s more open to judgment, I wouldn’t really know how they would be feeling and how their EDA would be changing. But I feel like **these videos are rather one-dimensional**, like this is just a cute dog. **There is only a small range of emotions that people can feel based on these videos**.” - B1

B1 is clear here that the only contextual information she had to judge EDA on was a cute dog video, from which she found herself unable to answer higher level questions about EDA as the context of understanding was so limited.

##### EDA as Persona

Beyond seeing EDA as associated with their own or their partner’s particular emotional experience, another common theme among participants was the interpretation of characteristics about a person from their EDA. Instances of this finding came up directly in interviews with 6 of the 9 pairs. In these cases, participants often spoke of learning more about “a new side” of their study partner that they didn’t have evidence of from other existing sources. G1 stated this when she described her thoughts on G2’s EDA response to a video of an impassioned climate change activist:

“I **had this image that [G2] is pretty calm and collected but then through what I see in his EDA from what’s shown in the video I could see that he can actually be affected really strongly by videos** which is interesting because maybe if I hadn’t seen that I wouldn’t have expected that of him [...] I think **I learned how you know in a way he’s very empathetic** and his emotions relates to what is conveyed in the video in a way” - G1

Here, G1 spoke to how specific activity in the EDA data occurring alongside particular events in the video content gave her insight into a piece of her friend’s persona. Much less frequent, but important instances of reading personal characteristics from EDA were those of participants concluding or questioning qualities about themselves from seeing their EDA information. Participants were sometimes surprised to see their EDA response, particularly in comparison to their partner’s, and in a few cases this resulted in questioning a currently held self-conception. D2, for example, stated:

“when I was looking at [my EDA], I would think oh you know this is emotional but mine didn’t change and I was like oh **maybe I’m not as empathetic towards their situation as I thought**” - D2

#### Participants’ Suggested Use Cases for EDA

Some final findings from the interviews worthy of note were participants’ own ideas for applications of socially sharing EDA and the next steps they felt would aid in their own understanding of EDA.

##### Suggestive yet Indirect

One concept generated by participants centered around using EDA to indicate and display the degree of emotional response in ways that are difficult to do with traditional forms of expressions like language. This in part relies on the aforementioned finding of seeing EDA as objective or true and thus a way to *prove* one’s feelings. D2 described how EDA could help in suggesting the level of their emotional state to their parents without providing specific detail:

“if I’m having an emotional conversation with my parents, **I’m not able to always describe what I’m feeling, there are things that I don’t want to bring up**, [...] anything that could go deeper, so I feel like it could be correlated that you would want them to understand some things, but in other ways they don’t need to know everything at once” - D2

In a similar vein, D1 described an idea to use EDA to help decide what to watch in a scenario where one or both people may not want to be direct with their opinions:

“I was thinking you could use it to determine whether or not you both like the show and would choose whether or not to watch that again together versus watch it individually. So **it could be a way to find something you both truly like** rather than someone saying “oh I really like this, would you watch it with me” but the other person is like “ehh I don’t really care” - D1

In both of these cases, EDA appears to be serving the role of direct communication at times when that communication may be difficult to bring oneself to engage in. While it is unclear if EDA would solve the problems posed by this scenario, it seems participants felt some utility in a sort of third party reporting of their emotional state. Related to this ambient display of emotion, the mention of wanting to see others’ EDA responses to scary movies came up in more than half of the group interviews. Fear in response to entertainment seemed to be an easily accessible example for participants to imagine EDA being shared. One participant spoke about wanting to use EDA to prove to their friends that they were significantly affected and thus didn’t want to watch scary movies. Others talked about the enjoyment of scary movies being tied to witnessing one another’s reactions to the tense and shocking moments, and that EDA could be a way to further this form of interpersonal sharing.

##### Invitation to Reflect & Talk

A common reaction to participants seeing activity in their partner’s EDA was not necessarily jumping to a conclusion about their partner’s specific feelings, but instead that it simply made them wonder, question, and want to ask their partner about how they were feeling. As H2 stated:

“ just like seeing it fluctuate and then when it did, like when it was higher or lower I was like **what was her reaction at this time, it just made me question**” - H2

Here H2 didn’t make a jump to how H1 was feeling when there was EDA activity, but rather it just made her wonder and question what it was about that moment that affected H1 in some way. This was a new experience for some participants, as talking about their feelings wasn’t commonplace, sometimes due to a lack of opportunities or triggers to do so. In fact, two participants mentioned therapy as a specific use case for EDA for its ability to display something unspoken and presumably talk about it with a therapist. In reflecting on what she had learned about her study partner during the study and why she hadn’t known this previously, G1 touched on a possible reason:

“I feel like in a way, we’ve only known each other for a year and **mostly we don’t really talk about like emotions in our conversations**, we tend to just talk about school and activities or memes or things that are funny, we don’t really have a lot of like heart to heart conversations about how we feel and yeah I think that could be a reason why we didn’t know this about each other.” - G1

For G1 and her study partner, EDA was a way to learn more about the emotional sides of one another that have otherwise been left unexplored. EDA alone didn’t constitute learning something about one’s partner however, instead it functioned as a trigger for the reflection and eventual conversation that helped improve knowing one another. D2 stated her sentiment that the conversation was crucial to understanding one another’s EDA response:

“I think like **a conversation would be needed** rather than just seeing the graphs because we could be feeling entirely different emotion but maybe some sort of an emotion shows up on the graph. But yeah **you really don’t know if it’s the same one so you would need a discussion**” - D2

This focus on reflection was also observed in a broader sense when participants compared their experience in the first session watching a video for the first time while their graph was simultaneously displayed, versus the second when watching the same video again with their partner’s EDA embedded. In the latter case, having already seen the content once themselves, participants could spend more time focusing on their partner’s EDA graph and reflecting on how the graph and video content were connected.

## Discussion

In studying the meaning-making process around sharing EDA, findings indicated the relevance of ecological and radical intersubjectivity approaches, that is that meanings can be formed through projecting one’s understanding of one’s own data onto another’s, and importantly also through dialogue about this new data type with close others. Many of the subtle triggers for conversation that can occur easily in free-flowing face-to-face conversation are absent in CMC systems, however a strength of EDA that emerged from this research was its function as a cue of *some* kind of emotional response and an invitation to talk more.

This research sought to provide answers to the two research questions posed early on, one about the experiences of physically distant friends and their perspectives on sharing EDA under these conditions, and another about how people form understandings of previously unknown biosensory information like EDA over time and via different exposures. While there were many compelling findings through this qualitative process, the discussion here is focused on these two research questions the study was designed to address.

### Unmet Needs of CMC and the Role of Sharing EDA

The COVID-19 pandemic, particularly its lasting presence in the US, forced many relationships that were once sustained by in-person interaction into a remote state using CMC technologies. By examining the experiences of the 18 participants in the present study, a better understanding of the effects this shift had on social dynamics can be gained and avenues for improvement revealed. The frustrations with CMC reported by study participants are not wholly new to CMC researchers, however the scenario of large-scale and long-term shift to CMC communication underscores their persistent drain on social well-being. The technological network and hardware issues participants spoke of have plagued CMC technologies since their inception, but of particular note here was the cumulative effect of issues like dropped connections and latency on participants’ motivation and willingness to reach out at all. This is concerning as social connection is necessary for human health and well-being and so the continued study and development of new fulfilling ways to communicate and share experiences is paramount.

From the online survey results it was observed that the bulk of formerly face-to-face interaction moved largely to video calling, though messaging also saw increased usage in this survey and in accounts from interviewees. In the interviews, both video calling and messaging suffered from unnaturalness and artificiality. Messaging was not held by participants as a way to have deep emotional conversations due to the effort involved in typing and the resulting challenge of having free-flowing conversations, nor did it help assuage the absence of spending time together. Video calling on the other hand is likely the “richest” media in widespread use - conveying audiovisual information of typically at least the face, in close to real-time. However, the physicality of in-person interactions was sorely missed by participants who engaged in video calling. The inability to touch one another and more so the lack of a shared interaction environment led to feelings of separation, lock-in, and anxiety provoked by a lack of awareness of one another’s other activities. A major appeal of social virtual reality systems for example is the regaining of social “presence”, which is achieved by gaining in the physicality of interaction with one another and the environment, but at the cost of representational fidelity and the clarity of other cues, e.g., facial expressions.

Perhaps one of the most intriguing findings was the pattern of participants reporting “just knowing” someone’s reaction to something shared via messaging or social media platforms. This seemed particularly relevant to sharing meme-like content, static images or short videos via platforms like Facebook and TikTok, due to the very nature of this form of media relying on shared experiences. In their work seeking to uncover why friends understand each other better than strangers, Colvin et al. and other empathic accuracy researchers suggest that friends develop an “intersubjective meaning context” which serves as an interpretive framework helping to better “read” one another’s emotions (Ickes, 1997; Hancock and Ickes, 1996). While these researchers were not studying CMC, interviewees’ accounts suggest this notion carries over to mediated communications as well. In CMC however, there are fewer cues or signals to contradict an assumption, which may happen no matter what duration or level of closeness a relationship has reached. This process is worth continued broad attention in CMC research, but the current study can speak to how social biosensing may fit into this landscape.

Can social biosensing help make up for any of the chasm that still separates in-person interaction from what is possible through CMC? The perspectives provided by the participants in this study can aid in answering this question. Similar to the existing practice of seeing a friend’s reaction to a piece of content via cues like facial expression and vocal qualities, this study examined participants’ processes and experiences interpreting reaction from EDA. None of the participants had previous knowledge of EDA, their own or otherwise, before participating in the study and so can be thought of as a simulation of new users of a social biosensing system. However, participants were all university undergraduate or graduate students in the US in their 20s and future research in this area should continue to expand the range of people, backgrounds, cultures, and beliefs that will surely affect the reception and utility of social biosensing systems.

A strength of EDA that emerged was its function as a cue of *some* kind of emotional response, and as an invitation to talk more. Many of the subtle triggers for conversation that can occur easily in free-flowing face-to-face conversation are absent in CMC systems. Of particular note in participant responses was the perception and desired usage of EDA as a “third-party” indicator. This status of the signal appeared to take some of the onus off of the individual, avoiding some of the vulnerability associated with speaking about one’s emotions. This vulnerability is often an essential part of developing trust and intimacy and should be wielded with caution. From the majority of participant perspectives EDA seemed to be of most valuable in close relationships where a significant amount of information about the other person is available. This seemed to allow participants to be more comfortable pushing past the ambiguity of the data and getting to know a close other in a way not even face to face interaction would allow for. Additionally the example scenario of watching scary movies with others came up in almost all interviews as a suggested use case, perhaps because this already involves a high degree of sharing and interpreting others’ reactions and thus social biosensing could enhance this existing practice.

On the whole EDA was seen as fairly authoritative by participants, even when the definition presented to them was left purposefully inexact and vague. Participants often searched their subjective experiences to explain something they saw in the EDA information, and it’s possible that at times they were made to believe they felt something they subjectively did not. Furthermore, participants not only read into emotions of the partner from their EDA, but also assigned *characteristics* to them based on the graph activity. These findings echo previous research indicating a tendency and at times even a desire people have to see biosensory information as objective, and the degree to which this influences perceived emotional states of oneself and others even when cautioned against the accuracy of this data for this purpose (Hollis et al., 2018; Howell et al., 2018). It is thus highly important that designers and developers think carefully about how to describe EDA information to users and for what contexts or purposes to use it in social biosensing systems. Once the data is in the hands of users however, people will form and update the meaning assigned to signals like EDA through their own experiences and conversation with others, both of which can be understood through the framework of intersubjectivity with its analogical and dialogical components.

### Intersubjective Meaning Construction & Empathy

Social biosensing is rife with interaction and meaning-making with data, often about signals that have no clear established meaning even among experts in the field. Future psychophysiological research may very well conclude that no matter the sophistication of the sensors or depth of preprocessing or analysis used, EDA and other biosignals do not have inherent, direct, or consistent connection to subjective inner states. This lays the groundwork for agency and creative interpretation in establishing meaning by users themselves of social biosensing systems. The question for social biosensing systems then becomes how does this process of meaning-making play out, which RQ2 of the present study sought to encapsulate.

Compared to other social biosensing related work, a major contribution of this study was the phased approach of data collection following exposure to one’s own, one’s partner’s, and finally discussion of EDA. The gathering of data at each of these time point allowed for the discovery of insights into the driving factors of the meaning-making process for an unfamiliar bodily signal between friends. The results of this inquiry make a strong case for the importance of intersubjectivity in the meaning-making process of biosensed data such as EDA. The changes in emotion association depicted in Figure 3 as well as the temporal meaning-making typologies and participant experiences described above are evidence of the degree of power social forces have in shaping users’ understanding of EDA.

Apperception, the act of perceiving more than what one directly observes using assumptions based on similar past experiences, such as perceiving the remaining structure of a building when one only sees the front side, relies on direct experience of an object or phenomenon. When it is applied to others the analogical component must be added as we are not able to directly perceive another’s full experience. Analogical apperception, a core mechanism of Crossley’s ecological intersubjectivity, is a useful conceptual lens to view social biosensing through, especially in situations when little other information is available. As highlighted in the present study and by participants’ interests in further experimentation with their own EDA data, similar past experiences with EDA aid in interpreting the EDA of another person. The “stablers” typology was also indicative of the occurrence of ecological intersubjectivity, as these participants formed and kept their understanding of EDA from their own experiences which they employed when interpreting their partner’s EDA.

Also evident in the present study however is the power of communication in the meaning-making process which emerged in the group interviews conducted after participants had viewed and assessed their own and their partner’s EDA in isolation. When examining participants’ meaning-making processes of EDA, the greatest shifts were observed when participants were able to discuss their impressions and thoughts with their partner. Crossley’s account of radical intersubjectivity posits that meaning is fully realized within an interaction and is irreducible into merely component parts from the individuals. The “broadeners”, and to some degree “puzzlers”, typologies uncovered in the results are indicative of sense-making occurring during an interaction, with the resulting derived meaning being made up of more than the two participants’ individual experiences. Despite the scientifically objective allure, signals like EDA are low precision indicators of internal states, and thus enabling and encouraging conversation about them in a social context is paramount to limiting possibly harmful misconceptions.

In the reality of the social world, Crossley concludes that we likely shift between ecological and radical intersubjectivity depending on the situation, and that both perspectives are required to account for the richness contained within social interactions. This framing shares many qualities with Weick’s collective sensemaking theory, which argues that sensemaking is an ongoing process shaped by the social environment, and that internal narratives are both individual and shared, evolving with conversations (Weick, 1995; Weick et al., 2005). Broadly, this theory describes sensemaking as a road to action which begs an important question of what people may *do* with the meanings they form about biosensory information. By regarding social biosensing through the lenses of intersubjectivity and sensemaking, HCI and CMC researchers can gain a stronger hold on the underlying social mechanisms and meaning formation taking place in this new way of connecting, and subsequently the actions that people may take rooted in these formed meanings.

### Research Limitations

Although fruitful, the research discussed here has several qualities which limit what can be interpreted or generalized. Firstly the diversity of the participant pool was limited in age, culture, and relationship types. All participants were in their 20’s and living in an urban environment in a large city in the western United States. All pairs were self-described close friends, none were family members, romantic partners, or in other relationship with one another. These factors limit the conclusions of this work to young friends in Western culture, and future work with more diverse audiences and relationship types would further elucidate possible experiences and social mechanisms in sharing biosensory information.

Another key limitation to this study is its duration over only a few sessions. While longer term use effects were not a goal of this study as designed, understanding longer term use is paramount to preparing for a world where social biosensing is widely available. Should social biosensing become more mainstream, signals like EDA will likely take on new meanings over longer periods of time as users discover the nuances of how their own EDA responds to various stimuli and scenarios, as well as how this compares to what they see from others sharing their EDA. Some social biosensing studies employing heart rate instead of EDA have tested systems over the course of a few weeks. For instance, Liu et al. conducted two studies based on heart rate sharing each over a 2-week duration, and many insights were garnered by naturally observing what situations arose over the course of the study period. Sharing as a way to lightly connect in moments of boredom, reduced sharing activity as the novelty wore off, or serendipitous moments that trigger interest in sharing one’s internal states like a difficult conversation with a loved one or the joy of a holiday celebration, were all enabled by longer term study of “in-the-wild” use (Liu et al., 2017b, 2019b).

### Implications by Audience

The results of this work have strong implications for philosophers and theorists, social biosensing researchers, and designers and developers of social computing systems. The value of the findings are best described in terms of each audience.

For the philosophers and theorists of intersubjectivity, this work expands the umbrella of possibilities to be considered under an intersubjective framework by methodically documenting CMC and social biosensing interactions. The core of intersubjectivity assumes in-person interpersonal relations, and while much of this is applicable to mediated communications, differences such as limited social and emotional cues, perceived artificiality, and the position of the device(s) powerfully shape interactions. The frustrations described by the study participants may very well be a result of technology’s interference with the radical intersubjective process, when free flowing conversational exchange is paramount. As for ecological intersubjectivity, if in-person empathy via analogical apperception can be thought of as imagining the rest of a house based on its front facade and previous experiences with houses, intersubjectivity through CMC and/or social biosensing may be more like attempting the same feat seeing only a pencil sketch of a house through a thick fog. To our knowledge this is the first study that has applied intersubjectivity to social biosensing, and as the findings showed strong evidence that the framework applies well, this should be merely opening the door to future work.

For those who will do this future work, the audience of social biosensing researchers, this study has important ramifications. The present study made it clear that participant perceptions of biosensory information in a social setting evolve over time, particularly with exposure to others’ data and through conversation. Researchers studying or evaluating new social biosensing systems should consider these dynamics in their study designs, analyses, and synthesis of results. This suggests the necessity of longer, multi-session, and ecologically valid time scales to gain a better understanding of perceptions and effects of social biosensing systems. Quantitative experimental researchers can push forward the methodology of this study which purposefully captured perceptions at different time points following specific stimuli. This technique employed with greater sample sizes and more representative populations would allow for further characterization of meaning-making patterns. In the realm of qualitative social biosensing research, group interviews have been regularly used to observe how people talk about their interpretations of social signalling with each other or how people interact in a scenario where their biosensory information is shared, but this primarily engages with the radical portion of intersubjectivity. To push our qualitative understandings of sense-making with social biosensing, ecological intersubjectivity can also be examined by gaining a deeper understanding of how meanings are formed based on individual experiences and then projected onto others via a process like analogical apperception. The “puzzlers” typology that emerged in this study, as well as those participants who did not fit into a typology, are indicative of the limitations of the present research where the meaning-making pattern was unclear, thus future researchers should interrogate and iterate on this framework.

And finally, this work can also offer some guidance for the designers and developers of these systems. As with researchers, designers can take into consideration the ecological and radical components of intersubjectivity by enabling comparisons between one’s own and others’ biosensory information for the former as well as stimulating conversation about this ambiguous data between interlocutors. Decisions around how to present unfamiliar signals like EDA, or even familiar yet misunderstood ones like heart rate, to users must be considered carefully. Even using a fairly ambiguous definition for EDA in common language in this study, some participants were convinced of its objective truth regarding inner states and even personality characteristics – dangerous assumptions for an arena as delicate as emotional disclosure and perception. Avoiding authoritative language or symbolism and encouraging questioning and contestation by signalling uncertainty are avenues to avoid this drawback. As discussed in this paper, biosensory information is quite inexact in how it relates to emotions as it is affected by many internal and external factors, and being transparent about these limitations (or even outright flagging them) is paramount to establishing user expectations. Users will also require time to form their own meanings of biosensory information through experimentation with their own and observation of others’, and internalize what can and cannot be gleaned from this form of data. Taking this into consideration for system design, including a certain amount of exploration in different contexts, with other people, and over time to users before more formal use would help allay the concerning possibility of users over-indexing on specific meanings too early. Furthermore, the visualization of EDA in this study was fairly basic, represented as a moving graph embedded into the video stimuli. There is a world of possibilities with regards to how EDA or other biosensory information could be represented, whether through other visual forms, or other sensory experiences like sound or haptics, some of which have been explored in other social biosensing research. When this data becomes visible or emphasized is also rife for design exploration, particularly if the decision of when to add emphasis or include data at all is placed into the hands of users to ensure agency over this private information is maintained.

## Conclusion

The global COVID-19 pandemic has shone a spotlight on the lacking nature of many dominant forms of computer-mediated communication, and underscored the need for new forms of emotive connection at a distance. This research explored social biosensing as one such method, focusing on the process of meaning-making about EDA information among physically separated friends, particularly through the lens of intersubjectivity. Through a phased exposure approach and individual as well as paired qualitative data, the importance of social factors in the meaning-making process became apparent. Though participants varied in their particular mode of temporal thinking, each engaged in a process well-represented through ecological or radical intersubjectivity when applying knowledge of their own EDA to understand a close other’s, and through dialogue. The import of new forms of connecting, whether through social biosensing or otherwise, while physically apart was brought to the forefront in participants’ recounting of frustrations with CMC when unable to interact in-person due to an ongoing global pandemic. Implications from this work apply to intersubjectivity theorists, social biosensing researchers, and HCI/CMC system designers and developers.
